# Translating Guidelines into Practice: A Prospective Real-World Study of a Romanian Cohort Treated with GLP-1 RAs

**DOI:** 10.3390/biomedicines13092174

**Published:** 2025-09-05

**Authors:** Mihaela Simona Popoviciu, Delia Reurean-Pintilei, Teodor Salmen, Marius Rus, Anca Ferician, Cristian Sava, Adriana Ioana Ardelean, Lavinia-Alexandra Moroianu, Anca Pantea Stoian

**Affiliations:** 1 Department of Diabetes, Nutrition and Metabolic Diseases-Clinical Section Internal Medicine I, Bihor County Emergency Clinical Hospital, 410169 Oradea, Romania; anca.ferician@uoradea.ro; 2 Department of Clinical Disciplines, Faculty of Medicine and Pharmacy, University of Oradea, 410073 Oradea, Romania; rusmarius@uoradea.ro; 3 Department of Medical-Surgical and Complementary Sciences, Faculty of Medicine and Biological Sciences, “Ștefan cel Mare” University, 720229 Suceava, Romania; delia.pintilei@usm.ro; 4 Doctoral School, “Carol Davila” University of Medicine and Pharmacy, 050474 Bucharest, Romania; Teodor.salmen@drd.umfcd.ro; 5 Pitesti Emergency County Hospital, 110283 Pitesti, Romania; 6 Department of Pediatrics, Emergency County Hospital Bihor, 410167 Oradea, Romania; cristian.sava@didactic.uoradea.ro; 7 Department of Medical Sciences, Faculty of Medicine and Pharmacy, University of Oradea, 410073 Oradea, Romania; 8 Department of Preclinical Sciences, Faculty of Medicine and Pharmacy, University of Oradea, 410073 Oradea, Romania; adriana_ardelean@uoradea.ro; 9 Department of Cardiology, Clinical Emergency County Hospital Oradea, 410169 Oradea, Romania; 10 Pharmaceutical Sciences Department, Dunarea de jos University, 800201 Galati, Romania; lavinia.moroianu@yahoo.com; 11 Diabetes, Nutrition and Metabolic Diseases Department, “Carol Davila” University of Medicine and Pharmacy, 050474 Bucharest, Romania; anca.stoian@umfcd.ro

**Keywords:** Type 2 diabetes mellitus, obesity, GLP-1 receptor agonists, semaglutide, exenatide, dulaglutide, glycemic control, real-world evidence

## Abstract

**Background/Objectives**: Obesity and type 2 diabetes mellitus (T2DM) have a continuously increasing prevalence and often co-exist, exacerbating cardiometabolic risk. GLP-1 receptor agonists (GLP-1 RAs) are recommended as first-line therapy for patients with T2DM and excess weight, particularly when cardiovascular risk is present. This study assessed the real-world effectiveness of available GLP-1 RAs in Romania on glycemic control, body weight reduction (BWR), and waist circumference (WC) in T2DM patients with excess weight. **Methods**: A prospective observational study was conducted on 311 adults with T2DM (glycated hemoglobin (HbA1c) > 7.2%, body mass index (BMI) ≥ 25 kg/m^2^). Patients received exenatide, semaglutide (either oral or injectable), or dulaglutide and were monitored for a period of 6 months. Parameters assessed included HbA1c, body weight, BMI, and WC. **Results**: All treatments significantly improved the patients’ HbA1c, BMI, and WC (*p* < 0.05). Dulaglutide had the most significant impact on HbA1c (−6.69 ± 0.91%), while injectable semaglutide led to the most notable BWR (−4.60 ± 2.74 kg) and WC reduction, especially among male patients. No significant differences in treatment effect were observed concerning the patient’s age, gender, or T2DM duration. **Conclusions**: In real-world clinical practice, GLP-1 RAs provide significant metabolic benefits and should be considered as part of individualized treatment strategies for T2DM patients who are overweight or obese.

## 1. Introduction

According to the World Health Organization (WHO), diabetes mellitus (DM) affects over 500 million people worldwide, with a significant increment in the estimation expected by mid-century, as supported by the WHO and recent global projections [1,2,3]. Obesity prevalence also has a positive trend, and is recognized as a central risk factor in the pathogenesis of DM [4,5,6,7]. In the current guidelines issued by the American Diabetes Association (ADA), the European Association for the Study of Diabetes (EASD), and the International Diabetes Federation (IDF), glucagon-like peptide-1 receptor agonists (GLP-1 RAs) are recommended as a first-line therapeutic choice in patients living with type 2 DM (T2DM) who are overweight or obese, particularly in the presence of increased cardiovascular (CV) risk [5,8]. For individuals living with established CV disease (CVD) or presenting major CV risk factors, the use of GLP-1 RAs is specifically advised [5,8]. These agents have shown significant efficacy not only in regard to improving glycemic control, but also in terms of promoting substantial body weight reduction (BWR) and CV risk reduction [5,9]. Furthermore, by addressing key components of metabolic syndrome, GLP-1 RAs contribute to a broader, integrated strategy for reducing overall CV morbidity and mortality in this high-risk population, including an increment in physical activity openness [10].

The present study provides real-world data from a Romanian cohort of patients with T2DM, whose management represents a major challenge, on effective and safe therapeutic interventions to reduce the risk of CV and metabolic complications [8,9,10,11,12,13,14]. Aligning with the most recent clinical guidelines and recommendations, which emphasize the importance of tailoring treatment strategies for attaining specific outcomes, such as glycemic control, BWR, and waist circumference (WC) reduction, a clear and comprehensive understanding of the therapeutic profiles of various GLP-1 RAs becomes increasingly important [8,9,10,15,16]. Given the heterogeneity among timely available GLP-1 RAs classes, which were approved in 2005, understanding the nuances of their efficacy and safety profiles and their impact on metabolic parameters is necessary in order to be able to provide individualized therapeutic strategies that meet the patients’ needs and improve the long-term outcomes [9,17]. Notably, these agents have consistently demonstrated their robustness and are comparable to basal insulin in terms of efficacy in improving glycemic control, promoting substantial BWR, and reducing CV risk [18,19]. Importantly, their long-standing clinical use over two decades has confirmed their favorable safety profile, with extensive data supporting both their metabolic benefits and their CV protection [18,19], so they are now being prescribed not only for managing T2DM, but also as frontline therapies for obesity and metabolic health [18,19,20].

Although the efficacy and safety of GLP-1 RAs have been well-established through randomized clinical trials, gathering data on their performance in everyday clinical practice remains essential [9,18,19,20]. Real-world evidence offers important insights into how these therapies work across various and broad patient populations and within the variability of diverse healthcare systems [9,19,20,21].

In this context, our study aimed to evaluate the effectiveness of different GLP-1 RA-based regimens in achieving metabolic control, specifically glycemic control, BWR, and WC improvements in patients with T2DM and various degrees of obesity under routine clinical conditions.

## 2. Materials and Methods

A prospective analysis was conducted on consecutively admitted patients who were overweight (defined as a body mass index (BMI) ≥ 25 kg/m^2^) or obese (defined as a BMI ≥ 30 kg/m^2^) and had T2DM, with suboptimal glycemic control (defined as glycated hemoglobin (HbA1c) > 7.2%). The study population was recruited consecutively from the inpatient Department in the Clinic Centre of Diabetes, Nutrition, and Metabolic Disorders at the Bihor County Emergency Hospital, Romania, over a period spanning from 22 November 2022 to 22 November 2023. The study protocol was developed in accordance with the ethical standards in the Declaration of Helsinki and received approval from the Institutional Ethics Committee at Bihor County Emergency Hospital (protocol number 25979, dated 21 November 2022). The study originally involved 333 consecutively included subjects. In the early days of data gathering, 10 subjects discontinued the assigned treatment regimens, whereas 12 more either refused to continue their participation after enrollment or could not complete the baseline assessments necessary for the final treatment evaluation, thus preventing them from being included in the final analysis.

Selection bias was prevented using the inclusion criteria that consisted of adult patients (>18 years of age) with a diagnosis of T2DM at least 6 months prior to enrolment, characterized by a HbA1c level of ≥ 7.2% and a BMI of ≥ 25 kg/m^2^ (overweight or obese). Patients had to be naive to GLP-1 RAs treatment, previously failed therapeutic schemes with other antidiabetic agents, and signed the informed consent form.

The exclusion criteria included patients: (1) with type 1 DM; (2) with a BMI < 25 kg/m^2^; (3) treated with insulin; (4) with a HbA1c < 7.2%; (5) with comorbidities, such as neoplasia, psychiatric or neurodegenerative disorders, medullary thyroid carcinoma or multiple endocrine neoplasia syndrome type 2, a history of pancreatitis, and severe gastrointestinal motility disorders, including gastroparesis; and (6) deemed to be ineligible in regard to the study objective.

The 311 included patients who received standard-of-care treatment, namely no insulin or pioglitazones, but a GLP-1 RA and when required any of metformin and/or sodium–glucose loop transporter 2, were distributed into four groups, according to the type of GLP-1 RA prescribed. In real-world Romanian clinical practice, the use of a semaglutide (both oral and injectable) slow titration scheme is secondary even due to a reimbursement restriction as part of the national healthcare system or due to the physician preference scheme, in order to improve the gastrointestinal tolerability of patients and, subsequently, patient adherence, so the dose variations used in this study are detailed in Table 1.

Patients were evaluated at the baseline and during 3-month and 6-month follow-up visits after treatment initiation, which included HbA1c, BMI, BWR, and WC assessments. The primary endpoint included HbA1c, while the secondary endpoint included BMI and WC.

Data processing was performed using SPSS, 20th edition, software and Microsoft Excel 2010 software. All the data are presented as the mean ± standard deviation (SD) for continuous variables and as numbers and percentages for categorical variables. Chi-square tests (χ^2^) were used to compare frequencies among the groups for categorical variables (e.g., patient gender). A *p*-value of less than 0.05 was considered statistically significant, with a 95% confidence interval (CI). Comparisons of the mean within groups for continuous variables (age, T2DM duration, HbA1c, BMI, BWR, and WC) were performed using analysis of variance (ANOVA). Where the ANOVA indicated significant differences, post hoc tests were conducted to determine specific differences between the groups. Sensitivity to change can be assessed in long-term observational studies. Changes in HbA1c, BMI, and WC were assessed over time, comparing the baseline values with those measured at the 3-month and 6-month follow-up visits. The effect size (ES) was calculated to evaluate the sensitivity to change, with ES estimates established according to standard rules for interpreting effect sizes (0.2—small, 0.5—medium, 0.8—large). The percentage reductions and mean changes in each group were calculated to illustrate the effects of different treatments on clinical outcomes. Midpoint reduction scores were calculated as the difference from the baseline to the 3-month follow-up and from the baseline to the 6-month follow-up. Multivariate linear and logistic regression were used to identify predictors for variable evolution. All the analyses were performed with a significance level set at α = 0.05, and 95% CIs were calculated to express the uncertainty of the estimates.

## 3. Results

Based on collected data on the group characteristics, Table 2 summarizes the comorbidities and T2DM comorbidities of the included patients.

The demographic characteristics of the groups regarding the patients’ gender, age, and T2DM duration are summarized in Table 3.

The evolution of HbA1c across the three visits of interest is summarized in Table 4.

Significant decreases were observed in the mean HbA1c values between the four groups at the baseline, at the 3-month visit, at the 6-month visit, and from the baseline within each group, as shown in Table 4.

Regardless of the type of treatment, the effect at 6 months on HbA1c was good or very good for each of the four groups, as shown in Table 4.

The changes in BMI across the three visits of interest are summarized in Table 5.

A statistically significant decrease in BMI was observed between the four groups at the baseline, 3 months and 6 months, and across all treatment groups, as shown in Table 5.

Regardless of the type of treatment, the effect on BMI at 6 months was minor for all four groups, as shown in Table 5.

The BWR changes over the three visits are summarized in Table 6.

At the 3-month follow-up visit, the BWR ranged from 1.29 kg in the exenatide injectable group to 1.90 kg in the semaglutide injectable group (*p* < 0.001). At the 6-month follow-up visit, the BWR ranged from 3.06 kg in the exenatide injectable group to 4.61 kg in the semaglutide injectable group (*p* < 0.001).

The reductions in WC by gender and across the follow-up visits are summarized in Table 7.

At the 3-month follow-up visit, the WC reduction ranged from 1.00 ± 0.38 cm in females treated with the exenatide injectable to 2.97 ± 1.29 cm in males treated with the semaglutide injectable. At the 6-month follow-up visit, the WC reduction ranged from 1.97 ± 0.84 cm in females treated with the exenatide injectable to 5.88 ± 2.89 cm in males treated with the semaglutide injectable. Significant within-group WC reductions were observed for all the therapies (*p* < 0.05).

The ES and percentage reductions in the patients’ WC between the follow-up visit are summarized in Table 8.

In the multivariate linear regression models, baseline HbA1c and injectable semaglutide use were strong independent predictors for a HbA1c evolution (R^2^ = 0.41, *p* < 0.001), while the male gender and a baseline BMI ≥ 35 kg/m^2^ were strong predictors for a WC reduction (*p* = 0.007).

In regard to the logistic regression, in order to obtain a BWR ≥ 5%, injectable semaglutide had an OR = 2.91 (95% CI 1.6–5.1, *p* < 0.001), while a T2DM duration < 5 years had an OR = 1.8 (95% CI 1.2–2.9, *p* = 0.022).

## 4. Discussion

This study confirms that GLP-1 RAs are effective in improving essential metabolic parameters across a diverse cohort of patients with T2DM and excess weight. Our findings are consistent with previous research [22,23,24,25,26,27,28,29,30,31,32,33,34] and validate the versatility of GLP-1 RAs in a real-world setting, outside the context of randomized controlled trials.

Our study population included a higher proportion of male patients, although gender differences were not statistically significant for most of the parameters (HbA1c, BMI, BWR, and WC). However, a WC reduction was significantly more pronounced in males, with a mean reduction of 6.51 cm compared to 4.13 cm in females at the 6-month evaluations. These gender-specific differences may reflect the underlying physiological differences in adipose tissue distribution, as males tend to accumulate a higher proportion of visceral adipose tissue that is more metabolically active and responsive to GLP-1RAs [26]. Our findings are consistent with previous research indicating that visceral adiposity is preferentially reduced by incretin-based therapies [22,23,24,25].

In our cohort, the metabolic response was not significantly impacted by age, despite a higher mean age in the dulaglutide group (62.38 ± 8.51 years) compared to the overall mean (59.85 ± 9.14 years). This aspect may reflect healthcare professionals’ preference for dulaglutide in older patients. While the literature suggests that GLP-1 RA tolerability may be decreased with age due to digestive tract-related side effects [27], the efficacy in terms of HbA1c and its impact on weight remains consistent across different age groups [28,29]. These findings are supported by our results and suggest that dulaglutide is an effective therapeutic option in older adults, with a low risk of hypoglycemia.

The T2DM duration ranged from 6 months to 27 years (mean 7.23 ± 5.09 years). Patients receiving injectable semaglutide or dulaglutide tended to have a longer disease duration (8.10 ± 4.83 years and 8.03 ± 6.49 years, respectively), possibly with a more advanced disease stage; however, even with the prolonged exposure to hyperglycemia, no significant attenuations in the HbA1c or BWR response were found, despite the existence of more severe beta cell dysfunction, insulin resistance, and limited physical mobility, due to complications [30]. This aspect suggests that the GLP-1 RAs retained their therapeutic efficacy in this clinical scenario.

Regarding complications, 83.3% of patients presented at least one T2DM complication, suggesting a high clinical burden due to prolonged inadequate metabolic control. The prevalence of HBP (65.3%), CVD (59.2%), and dyslipidemia (43.7%) reflects the need for novel beneficial CV therapeutic agents, such as GLP-1 RAs and SGLT-2i [8].

The baseline HbA1c values (>8%) were above the recommended therapeutic target, aligning with other reports of therapeutic inertia, defined as the failure to intensify treatment in a timely manner, despite suboptimal control [23,34,35,36,37,38,39,40]. Our findings emphasize the need for the application of early guideline-recommended [36] high-efficacy therapies, such as GLP-1 RAs, to reduce the risk of complications of the micro- and macro-vasculature [37,38].

Our data shows that the HbA1c levels significantly decreased across all four treatment groups, regardless of the medication used. At both the 3-month and 6-month follow-up visits, the HbA1c reductions were statistically significant (*p* < 0.05), reflecting improved metabolic control. Injectable semaglutide recorded the largest percentage reduction in HbA1c at 6 months (−21.36%), followed by oral semaglutide (−18.54%) and dulaglutide (−17.42%) [41]. Oral semaglutide significantly reduced HbA1c (−18.54% at 6 months), providing an effective option for patients who prefer oral administration. Exenatide and dulaglutide showed efficacy in reducing HbA1c, but with a larger SD, indicating greater patient response variability. The statistical effect (effect size—ES) was moderate to large at 6 months, above 0.8 for exenatide (1.274) and oral semaglutide (1.14), respectively, indicating a substantial treatment impact. At the 6-month follow-up visit, the greatest HbA1c reduction was observed in the dulaglutide group (6.69 ± 0.89), followed by the oral and injectable semaglutide groups. The higher efficacy of GLP-1 RAs in reducing HbA1c, often superior to other drug classes, has been supported by previous studies, such as that by Aroda et al. [42], demonstrating the robust benefits of semaglutide, both as a monotherapy and in combination with other antidiabetic therapies [43,44]. Similarly, another study reported significant HbA1c reductions in patients treated with dulaglutide, with a favorable safety profile [45]. Also, in the SUSTAIN 7 and SUSTAIN 10 studies, a superior reduction in HbA1c and body weight was reported for semaglutide versus exenatide or dulaglutide [46,47]. In our study, for injectable semaglutide, a 21.36% reduction in HbA1c, with a mean 4.61 kg BWR, align with the findings from the PIONEER trials [48]. What is more, over 73% of patients achieved a ≥ 1% reduction in HbA1c, with 81.7% in the injectable semaglutide group. Although exploratory analyses suggest a trend towards greater reductions in HbA1c and BWR with higher semaglutide doses, these differences did not reach statistical significance during the 6-month observation period. The lack of a clear dose–response effect may be related to the relatively short follow-up period and the small sample size in our study.

An important finding, considering the well-established association between a lower BMI and the reduction of risk for major CV and metabolic complications in patients with T2DM, is the effect on BMI. Despite sustained global efforts, obesity prevalence continues to rise, and no country within the European Union or the United States has successfully reversed this trend [49,50,51,52]. Multiple studies have confirmed the efficacy of GLP-1 RAs in achieving sustained BWR and improving weight control [23,24,53,54,55,56,57,58,59]. Our data reveals that oral semaglutide demonstrated efficacy in reducing the patients’ BMI, supporting its role as an alternative to injectable therapies, even though it is associated with a slightly lower BWR compared to the injectable formulation, as per previous reports [60]. Dulaglutide significantly reduced the patients’ BMI and WC, confirming its effectiveness in terms of weight control. These results are consistent with the AWARD-6 study, showing similar metabolic improvements [45]. The reduction in BMI was consistent across the treatments, improving metabolic control and CV risk in the patients. These real-world data support the role of GLP-1 RAs in managing T2DM and obesity by sustaining BWRs and enhancing metabolic outcomes.

Patients with a higher baseline BMI, such as those in the oral semaglutide group, required a longer duration to achieve a meaningful BWR and reductions in their WC. In contrast, participants receiving injectable semaglutide or dulaglutide exhibited more rapid improvements, potentially due to differences in obesity severity or the pharmacological limitations of oral semaglutide, which requires strict patient adherence to the dosing instructions (including fasting administration, a fixed water quantity, and delayed food and liquid intake). Although no statistically significant differences between the treatment groups were evident at the 6-month follow-up visit, longer-term follow-up might reveal more distinct outcomes based on the patients’ baseline BMI. At the 3-month follow-up visit, the greatest BWR was observed in the injectable semaglutide group (1.94 ± 0.95 kg), and this trend persisted at the 6-month follow-up visit. These real-world findings are consistent with published evidence [61,62,63]. Dulaglutide and oral semaglutide resulted in a slightly lower, yet comparable, BWR to injectable semaglutide, aligning with the recent systematic review by Singh AK et al. on oral semaglutide efficacy in T2DM [64]. The continued BWR observed between the 3-month and 6-month follow-up visits reflects the sustained metabolic benefits of GLP-1 RAs from ongoing treatment, as demonstrated in other studies [44].

Significant intergroup differences in regard to the WC reduction were also noted, with relevant gender-based variations. In the injectable semaglutide group, males experienced the most pronounced reductions, from 2.97 ± 1.29 cm at the 3-month follow-up visit to 5.88 ± 2.89 cm at the 6-month follow-up visit, most likely due to a higher proportion of visceral fat, which is more responsive to GLP-1 RA therapy [22,45,65,66,67,68]. Among the female participants, injectable semaglutide again led to the greatest WC reduction (2.02 ± 1.17 cm at the 3-month follow-up visit and 3.80 ± 1.79 cm at the 6-month follow-up visit), followed by dulaglutide and oral semaglutide. Although oral semaglutide showed slightly smaller effects, it remains a valuable alternative for patients who prefer oral therapy. Dulaglutide performed comparably to injectable semaglutide, while exenatide demonstrated the least effect, potentially limiting its use in patients with marked abdominal obesity. These findings are similar to the evidence linking both BWR and WC reduction to decreased CV risk in individuals with T2DM [3,22,69,70].

Moreover, 58% of patients lost ≥ 5% of their baseline weight, reaffirming GLP-1 RAs role in weight management in T2DM. Interestingly, patients with a longer T2DM duration (≥ 5 years) had a higher response rate in regard to both HbA1c and BWR. This may be due to their higher baseline glycemic values or prior treatment burden, increasing the relative impact of GLP-1 RAs. In contrast, male patients showed greater reductions in WC, consistent with known gender differences in visceral fat distribution and metabolic responsiveness [71]. These observations emphasize the importance of personalized treatment strategies that consider baseline characteristics, such as gender, T2DM duration, and body composition.

The superior performance of injectable semaglutide may be attributed to its higher GLP-1 receptor affinity, longer half-life, and central appetite-suppressing effects [28]. Although oral semaglutide is a promising alternative, its bioavailability is significantly lower, which may explain the comparatively smaller, although still meaningful, reductions observed.

### Comparison with Recent Real-World Evidence

Our findings align with those by Caruso et al. [72], who, in a retrospective cohort phenotyped by Ahlqvist/Bello-Chavolla algorithms, reported that higher baseline HbA1c levels are a dominant predictor of HbA1c reductions due to GLP-1 RAs therapy, with severe insulin-deficient DM patients exhibiting larger absolute declines; however, the phenotype effect attenuated after the adjustment for baseline HbA1c. Consistent with this result, in our cohort, baseline HbA1c and the use of injectable semaglutide were independent predictors of glycemic improvement at 6 months. In contrast, Marassi et al. [73], reported a greater long-term BWR in females as compared to males across GLP-1 RA therapy in a large, multicentric Italian cohort (*n* = 7800), independent of dosing. Our gender-stratified analysis focused on WC and showed a larger BWR in males at 6 months, while the HbA1c reductions were broadly similar for both genders. Differences in the outcomes, BWR versus WC, follow-up duration; multiple years versus 6 months, cohort size; multicenter versus single-center, and baseline profiles; and BMI distribution and comorbidity burden, likely account for the heterogenous gender-related patterns.

In terms of a comparison with RCTs, our findings mirror their results in terms of glycemic control, BWR, and abdominal fat reduction. Nevertheless, unlike trials with strict inclusion criteria, our study captures a more heterogeneous and comorbid population, with different demographic backgrounds. This real-world evidence highlights the external validity of GLP-1 RA therapy and supports its application across diverse patient profiles. Notably, studies such as SUSTAIN-6 and PIONEER-6 demonstrated the CV benefits of semaglutide, while the AWARD studies confirmed the efficacy of dulaglutide. Our results reflect comparable outcomes, validating the use of these agents during typical clinical practice, including among older adults and those with long-standing T2DM.

Beyond its statistical significance, the magnitude and the pattern of change that were observed here are clinically relevant. The HbA1c improvements align with large, randomized control trial results for semaglutide and dulaglutide, while the WC reduction, which is more pronounced in males, most likely reflects gender-specific visceral fat physiology. The differences as compared to longer and larger, multicenter real-world cohorts relate most probably to follow-up lengths, baseline risk factors, and the strategy of titration. In routine clinical practice, reimbursement constraints and tolerability-driven slow titration can attenuate the apparent dose–response, but may also enhance persistence. Our data support the early, guideline-directed use of GLP-1 RAs in patients with T2DM with excess weight, with attention to gender, the patient’s HbA1c at the baseline, and adherence as key factors.

Several limitations need to be acknowledged: (1) our observational, non-randomized design limits causal inference; (2) the relatively small sample size may reduce the power for subgroup analyses; (3) the short follow-up period (6 months) prevents the assessment of long-term outcomes; (4) treatment adherence, especially relevant for oral semaglutide, was not objectively assessed; (5) adverse events, which may influence tolerability and persistence, were not systematically captured; (6) lifestyle and dietary factors, although with a known and powerful impact, were not standardized or controlled; (7) a lack of assessment of fasting glucose and insulin levels or HOMA-IR; and (8) a lack of T2DM cluster phenotyping.

Despite these limitations our study presents several notable strengths. We prospectively conducted this analysis in a real-world clinical setting by using strict inclusion and exclusion criteria to ensure the selection of a clinically relevant and homogeneous population, namely adults who were overweight or obese, inadequately controlled T2DM (HbA1c > 7.2%), and no prior exposure to GLP-1 RAs. Importantly, the sample was consecutively recruited, which minimized selection bias. All the patients were systematically evaluated at the baseline, 3-month follow-up, and 6-month follow-up visits, using consistent metrics, namely HbA1c, BMI, body weight, and WC, which were analyzed using validated statistical methods. The ES calculations (Cohen’s d) were employed to assess the magnitude of change over time, providing interpretable data from a clinical point of view, beyond statistical significance. The study also included a gender-stratified analysis of WC reduction, which provides a glimpse into gender-based variability in the treatment response, an area insufficiently explored in the current literature.

The consistent improvements in HbA1c, BMI, and WC across all the GLP-1 RA formulations parallel those reported in randomized control trials, but reflect routine clinical setting care conditions, where treatment decisions are influenced by patient preferences, behavior, comorbidities, and drug tolerability. To the best of our knowledge, this is the first prospective real-world study assessing GLP-1 RA effectiveness in a Romanian population under routine clinical conditions. This real-world applicability, combined with stratified analyses and ES estimates, enhances the external validity of our findings and supports the broader clinical use of GLP-1 RAs in diverse patient populations. In future phases, following this analysis, the focus will include longer term outcomes and stratified analyses according to the baseline obesity class, comorbidities, and treatment persistence of patients to inform more personalized therapeutic strategies.

## 5. Conclusions

This real-world study confirms the effectiveness of GLP-1 RAs in improving glycemic control, BWR, and lowering the WC in patients with T2DM and excess weight, with consistent benefits observed across genders, ages, and disease durations, with a greater abdominal fat reduction in males. To the best of our knowledge, this is the first real-world prospective analysis of GLP-1 RA use in a Romanian cohort. These findings support the early integration of GLP-1 RAs into standard care, in alignment with current guidelines, and underscore the need for larger, multicenter studies to guide personalized therapy and optimize comorbidity management through multidisciplinary care.

## Figures and Tables

**Table 1:** Patient distribution according to the GLP-1 RA type prescribed.

Group	Treatment	Dose Prescribed	Number of Patients
1	Exenatide injectable (ow)	2 mg	17
2	Semaglutide injectable (ow)	0.25 mg	23
		0.5 mg	70
		1 mg	16
3	Semaglutide oral (od)	3 mg	92
		7 mg	12
		14 mg	10
4	Dulaglutide injectable (ow)	1.5 mg	71

ow—once weekly; od—once daily.

**Table 2:** Comorbidities and T2DM complications of the included patients.

Comorbidities/T2DM Complications	Number of Patients, Percentage
High Blood Pressure	203, (65.3)
Cardiovascular Disease	184, (59.2)
Dyslipidemia	136, (43.7)
Chronic Kidney Disease	30, (9.6)
Peripheral Neuropathy	145, (53.1)
Amputation below the ankle	5, (1.6)
≥1 T2DM Complications	259, (83.3)

T2DM—Type 2 Diabetes Mellitus.

**Table 3:** Demographic characteristics of the study groups regarding the patients’ gender, age, and T2DM duration.

	Gender (Female/Male)	*p*-Value
Exenatide injectable	7 (41.2%)/10 (58.8%)	0.186
Semaglutide injectable	46 (42.2%)/63 (57.8%)	
Semaglutide oral	46 (40.4%)/68 (59.6%)	
Dulaglutide injectable	32 (45.1%)/39 (54.9%)	
	**Age (37–78 years)**	***p*-Value**
Exenatide injectable	57.29 ± 7.29 years	0.069
Semaglutide injectable	59.36 ± 9.97 years	
Semaglutide oral	59.37 ± 9.28 years	
Dulaglutide injectable	62.38 ± 8.51 years	
	**Duration of T2DM (6 months–27 years)**	***p*-Value**
Exenatide injectable	6.91 ± 2.52 years	0.040
Semaglutide injectable	8.10 ± 4.83 years	
Semaglutide oral	6.51 ± 2.74 years	
Dulaglutide injectable	8.03 ± 6.49 years	

Data are presented as number (%) for categorical variables and mean ± SD for continuous variables. T2DM—Type 2 Diabetes Mellitus; SD—standard deviation.

**Table 4:** The mean (%) variation, ES, and percentual reduction in HbA1c between the visits.

	Baseline	3-Month Visit	6-Month Visit	Mean 95% CI, *p*-Value	3-Month ES	6-Month ES	3-Month Reduction (%)	6-Month Reduction (%)
Exenatide injectable	8.47 ± 1.09	7.69 ± 1.14	7.09 ± 1.11	95% CI (7.40, 8.09), *p* = 0.003	0.72	1.274	−9.22	−16.30
Semaglutide injectable	8.99 ± 2.00	7.71 ± 1.30	7.07 ± 0.96	95% CI (7.74, 8.11), *p* < 0.001	0.64	0.96	−14.24	−21.36
Oral semaglutide	8.83 ± 1.44	7.92 ± 0.88	7.19 ± 0.61	95% CI (7.85, 8.11), *p* < 0.001	0.63	1.14	−10.27	−18.54
Dulaglutide injectable	8.10 ± 1.79	7.25 ± 1.231	6.69 ± 0.89	95% CI (7.5, 7.55), *p* < 0.001	0.58	0.79	−10.50	−17.42
Mean 95% CI, *p*-value	95% CI (8.50, 8.892), *p* = 0.006	95% CI (7.55, 7.81), *p* = 0.002	95% CI (6.94, 7.13), *p* = 0.001					

CI—confidence interval; ES—effect size.

**Table 5:** The mean (kg/m^2^) reduction, ES, and percentage variation in BMI between the visits.

	Baseline	3-Month Visit	6-Month Visit	Mean 95% CI, *p*-Value	3-Month ES	6-Month ES	3-Month Reduction (%)	6-Month Reduction (%)
Exenatide injectable	33.73 ± 3.53	33.12 ± 3.68	32.43 ± 3.54	95% CI (32.10, 34.11), *p* = 0.575	0.17	0.37	−1.81	−3.85
Semaglutide injectable	33.43 ± 3.55	32.78 ± 5.38	31.78 ± 4.98	95% CI (32.38, 34.49), *p* = 0.070	0.12	0.30	−1.94	−4.94
Oral semaglutide	34.95 ± 5.35	34.19 ± 5.36	33.75 ± 5.30	95% CI (33.80, 34.94), *p* = 0.236	0.10	0.22	−2.17	−3.43
Dulaglutide injectable	32.61 ± 6.70	32.04 ± 6.69	31.30 ± 6.65	95% CI (31.08, 32.88), *p* = 0.502	0.09	0.20	−1.75	−4.02
Mean 95% CI, *p*-value	95% CI (33.20, 34.46), *p* = 0.043	95% CI (32.60, 33.86), *p* = 0.032	95% CI (31.81, 33.04), *p* = 0.011					

CI—confidence interval; ES—effect size; BMI—body mass index.

**Table 6:** The mean BWR (kg) between the visits.

	Baseline	3-Month Visit	6-Month Visit	Mean 95% CI, *p*-Value	3-Month ES	6-Month ES	3-Month Reduction (%)	6-Month Reduction (%)
Exenatide injectable	83.24 ± 7.64	81.94 ± 7.54	80.18 ± 6.98	95% CI (79.1, 83.85), *p* = 0.485	0.17	0.40	−1.55	−3.67
Semaglutide injectable	86.13 ± 15.61	84.23 ± 15.34	81.51 ± 14.30	95% CI (82.30, 85.60), *p* = 0.078	0.12	0.30	−2.20	−5.36
Semaglutide oral	95.56 ± 11.75	94.08 ± 11.79	92.26 ± 11.73	95% CI (92.71, 95.22), *p* = 0.107	0.13	0.28	−1.55	−3.45
Dulaglutide injectable	89.79 ± 15.99	88.23 ± 16.03	86.15 ± 15.88	95% CI (85.90, 90.21), *p* = 0.398	0.10	0.23	−1.74	−4.05
Mean 95% CI, *p*-value	95% CI (88.62, 91.90), *p* < 0.001	95% CI (88.99, 90.25), *p* < 0.001	95% CI (84.89, 88.03), *p* < 0.001					

BWR—body weight reduction; CI—confidence interval.

**Table 7:** The mean WC (cm) reduction between the visits.

	Females		Males		*p*-Value
	3-Month Visit (cm)	6-Month Visit (cm)	3-Month Visit (cm)	6-Month Visit (cm)	
Exenatide injectable	1.00 ± 0.38	1.97 ± 0.84	2.65 ± 1.77	4.29 ± 1.26	0.004
Semaglutide injectable	2.02 ± 1.17	3.80 ± 1.79	2.97 ± 1.29	5.88 ± 2.89	<0.001
Semaglutide oral	1.71 ± 0.91	2.93 ± 1.10	2.56 ± 1.04	4.12 ± 1.34	<0.001
Dulaglutide injectable	1.66 ± 0.48	3.43 ± 0.79	2.67 ± 1.34	4.73 ± 1.74	<0.001
*p*-value	0.176	0.072	0.708	0.062	

WC—waist circumference.

**Table 8:** The ES and percentage reduction in the WC (cm) of the patients between the visits.

	ES				Percentage reduction			
	Females		Males		Females		Males	
	3-Month Visit	6-Month Visit	3-Month Visit	6-Month Visit	3-Month Visit	6-Month Visit	3-Month Visit	6-Month Visit
Exenatide injectable	0.08	0.16	0.40	0.70	−1.12	−2.21	−3.02	−4.90
Semaglutide injectable	0.12	0.23	0.19	0.37	−2.20	−4.13	−3.29	−6.51
Oral semaglutide	0.11	0.19	0.23	0.37	−1.84	−3.16	−2.59	−4.04
Dulaglutide injectable	0.08	0.16	0.23	0.1	−1.84	−3.82	−2.70	−4.78

ES—effect size; WC—waist circumference.

## Data Availability

The data are available on request from the authors, due to the fact that this study is part of a larger study that intends to publish its results in the future.

## Abbreviations

The following abbreviations are used in this manuscript:

ADA	American Diabetes Association
BMI	Body mass index
BWR	Body weight reduction
CI	Confidence interval
CV	Cardiovascular
CVD	Cardiovascular disease
DM	Diabetes mellitus
EASD	European Association for the Study of Diabetes
ES	Effect size
GLP-1 RAs	Glucagon-like peptide-1 receptor agonists
IDF	International Diabetes Federation
SD	Standard deviation
T2DM	Type 2 diabetes mellitus
WC	Waist circumference
WHO	World Health Organization

## References

[1] World Health Organization Diabetes. https://www.who.int/news-room/fact-sheets/detail/diabetes.

[2] Halsey G. (2023). Global Diabetes Prevalence Will Double by 2050, Affecting 1.3 Billion People: New Predictions.

[3] Ahrițculesei R.-V., Boldeanu L., Vladu I.M., Clenciu D., Mitrea A., Cîmpeanu R.C., Mustață M.-L., Siloși I., Boldeanu M.V., Vere C.C. (2024). Correlation Between Prognostic Nutritional Index, Glasgow Prognostic Score, and Different Obesity-Related Indices in People with Diabetes or Prediabetes. Diagnostics.

[4] Alam S., Hasan M.K., Neaz S., Hussain N., Hossain M.F., Rahman T. (2021). Diabetes Mellitus: Insights from Epidemiology, Biochemistry, Risk Factors, Diagnosis, Complications and Comprehensive Management. Diabetology.

[5] Salmen T., Pietrosel V.-A., Reurean-Pintilei D., Iancu M.A., Cimpeanu R.C., Bica I.-C., Dumitriu-Stan R.-I., Potcovaru C.-G., Salmen B.-M., Diaconu C.-C. (2024). Assessing Cardiovascular Target Attainment in Type 2 Diabetes Mellitus Patients in Tertiary Diabetes Center in Romania. Pharmaceuticals.

[6] Chandrasekaran P., Weiskirchen R. (2024). The Role of Obesity in Type 2 Diabetes Mellitus—An Overview. Int. J. Mol. Sci..

[7] Cîmpeanu R.-C., Caragea E.-M., Mustață L.-M., Forțofoiu D., Dragne I.-G., Alexa R.-E., Balta A., Ceasovschih A., Șorodoc L., Săndulescu L.-D. (2025). The Involvement of Serotonin in the Obesity Pathway—A Last Decade Systematic Review of the Literature. Int. J. Mol. Sci..

[8] (2025). American Diabetes Association Professional Practice Committee. 9. Pharmacologic Approaches to Glycemic Treatment: Standards of Care in Diabetes—2025. Diabetes Care.

[9] Salmen T., Rizvi A.A., Rizzo M., Pietrosel V.-A., Bica I.-C., Diaconu C.T., Potcovaru C.G., Salmen B.-M., Coman O.A., Bobircă A. (2023). Antidiabetic Molecule Efficacy in Patients with Type 2 Diabetes Mellitus—A Real-Life Clinical Practice Study. Biomedicines.

[10] Nasui B.A., Talaba P., Nasui G.A., Sirbu D.M., Borda I.M., Pop A.L., Ciortea V.M., Irsay L., Purcar-Popescu A.I., Cinteza D. (2022). The Influence of Diet and Physical Activity on Oxidative Stress in Romanian Females with Osteoarthritis. Nutrients.

[11] Khan M.A.B., Hashim M.J., King J.K., Govender R.D., Mustafa H., Al Kaabi J. (2020). Epidemiology of Type 2 Diabetes—Global Burden of Disease and Forecasted Trends. J. Epidemiol. Glob. Health.

[12] Ruze R., Liu T., Zou X., Song J., Chen Y., Xu R., Yin X., Xu Q. (2023). Obesity and type 2 diabetes mellitus: Connections in epidemiology, pathogenesis, and treatments. Front. Endocrinol..

[13] Bohiltea R.E., Ducu I., Mihai B.M., Iordache A.-M., Dima V., Vladareanu E.M., Bacalbasa N., Bohiltea A.-T., Salmen T., Varlas V. (2022). First-Trimester Diagnosis of Supernumerary Hemivertebra. Diagnostics.

[14] Powell-Wiley T.M., Poirier P., Burke L.E., Després J.P., Gordon-Larsen P., Lavie C.J., Lear S.A., Ndumele C.E., Neeland I.J., Sanders P. (2021). American Heart Association Council on Lifestyle and Cardiometabolic Health; Council on Cardiovascular and Stroke Nursing; Council on Clinical Cardiology; Council on Epidemiology and Prevention; and Stroke Council. Obesity and Cardiovascular Disease: A Scientific Statement From the American Heart Association. Circulation.

[15] Popoviciu M.S., Salmen T., Reurean-Pintilei D., Voiculescu V., Pantea Stoian A. (2025). SGLT-2i—A Useful Tool for Real-Life Metabolic and Body Weight Control in Type 2 Diabetes Mellitus Patients. Medicina.

[16] Opriș-Belinski D., Cobilinschi C.O., Săulescu I. (2024). Current Perspectives in Giant Cell Arteritis: Can We Better Connect Pathogenesis and Treatment?. Medicina.

[17] Chan V., Cao L., Wong M.M.H., Lo K., Tam W. (2023). Diagnostic accuracy of waist-to-height ratio, waist circumference, and body mass index in identifying metabolic syndrome and its components in older adults: A systematic review and meta-analysis. Curr. Dev. Nutr..

[18] Ng E., E Shaw J., Wood A., Maple-Brown L.J., Hare M.J. (2022). Glucagon-like peptide-1 receptor agonist (GLP1-RA) therapy in type 2 diabetes. Aust. J. Gen. Pr..

[19] Marx N., Husain M., Lehrke M., Verma S., Sattar N. (2022). GLP-1 receptor agonists for the reduction of atherosclerotic cardiovascular risk in patients with type 2 diabetes. Circulation.

[20] Jensterle M., Rizzo M., Haluzík M., Janež A. (2022). Efficacy of GLP-1 RA approved for weight management in patients with or without diabetes: A narrative review. Adv. Ther..

[21] Schneeweiss S., Patorno E. (2021). Conducting real-world evidence studies on the clinical outcomes of diabetes treatments. Endocr. Rev..

[22] Marso S.P., Bain S.C., Consoli A., Eliaschewitz F.G., Jódar E., Leiter L.A., Lingvay I., Rosenstock J., Seufert J., Warren M.L. (2016). SUSTAIN-6 Investigators. Semaglutide and Cardiovascular Outcomes in Patients with Type 2 Diabetes. N. Engl. J. Med..

[23] Wilding J.P.H., Batterham R.L., Calanna S., Davies M., Van Gaal L.F., Lingvay I., McGowan B.M., Rosenstock J., Tran M.T.D., Wadden T.A. (2021). STEP 1 Study Group. Once-Weekly Semaglutide in Adults with Overweight or Obesity. N. Engl. J. Med..

[24] Wadden T.A., Bailey T.S., Billings L.K., Davies M., Frias J.P., Koroleva A., Lingvay I., O’neil P.M., Rubino D.M., Skovgaard D. (2021). STEP3 Investigators Effect of Subcutaneous Semaglutide vs Placebo as an Adjunct to Intensive Behavioral Therapy on Body Weight in Adults With Overweight or Obesity: The STEP3 Randomized Clinical Trial. JAMA.

[25] Rubino D., Abrahamsson N., Davies M., Hesse D., Greenway F.L., Jensen C., Lingvay I., Mosenzon O., Rosenstock J., Rubio M.A. (2021). STEP4 Investigators Effect of Continued Weekly Subcutaneous Semaglutide vs Placebo on Weight Loss Maintenance in Adults With Overweight or Obesity: The STEP4 Randomized Clinical Trial. JAMA.

[26] Jensen M.D., Ryan D.H., Apovian C.M., Ard J.D., Comuzzie A.G., Donato K.A., Hu F.B., Hubbard V.S., Jakicic J.M., Kushner R.F. (2014). 2013 AHA/ACC/TOS guideline for the management of overweight and obesity in adults: A report of the American College of Cardiology/American Heart Association Task Force on Practice Guidelines and The Obesity Society. Circulation.

[27] Du Y.F., Ou H.Y., Beverly E.A., Chiu C.J. (2014). Achieving glycemic control in elderly patients with type 2 diabetes: A critical comparison of current options. Clin. Interv. Aging.

[28] Nauck M.A., Quast D.R., Wefers J., Meier J.J. (2021). GLP-1 receptor agonists in the treatment of type 2 diabetes—State-of-the-art. Mol Metab..

[29] Gilbert M.P., Pratley R.E. (2020). GLP-1 Analogs and DPP-4 Inhibitors in Type 2 Diabetes Therapy: Review of Head-to-Head Clinical Trials. Front. Endocrinol..

[30] Ahmann A.J., Capehorn M., Charpentier G., Dotta F., Henkel E., Lingvay I., Holst A.G., Annett M.P., Aroda V.R. (2018). Efficacy and Safety of Once-Weekly Semaglutide Versus Exenatide ER in Subjects With Type 2 Diabetes (SUSTAIN 3): A 56-Week, Open-Label, Randomized Clinical Trial. Diabetes Care.

[31] Campbell R.K. (2011). Clarifying the role of incretin-based therapies in the treatment of type 2 diabetes mellitus. Clin. Ther..

[32] Lenharo M. (2024). Obesity-drug pioneers win prestigious Lasker Award for medical science. Nature.

[33] Knudsen L.B. (2024). GLP-1 for Treating Obesity-Origin, History, and Evolution: 2024 Lasker-DeBakey Clinical Medical Research Award. JAMA.

[34] Shaefer C.F., Kushner P., Aguilar R. (2015). User’s guide to mechanism of action and clinical use of GLP-1 receptor agonists. Postgrad. Med..

[35] Saydah S.H., Fradkin J., Cowie C.C. (2004). Poor control of risk factors for vascular disease among adults with previously diagnosed diabetes. JAMA.

[36] (2024). American Diabetes Association Professional Practice Committee. 6. Glycemic Goals and Hypoglycemia: Standards of Care in Diabetes—2024. Diabetes Care.

[37] Zoungas S., Arima H., Gerstein H.C., Holman R.R., Woodward M., Reaven P., Hayward R.A., Craven T., Coleman R.L., Chalmers J. (2017). Effects of intensive glucose control on microvascular outcomes in patients with type 2 diabetes: A meta-analysis of individual participant data from randomised controlled trials. Lancet Diabetes Endocrinol..

[38] Ismail-Beigi F., Craven T., Banerji M.A., Basile J., Calles J., Cohen R.M., Cuddihy R., Cushman W.C., Genuth S., Grimm R.H. (2010). ACCORD trial group. Effect of intensive treatment of hyperglycaemia on microvascular outcomes in type 2 diabetes: An analysis of the ACCORD randomised trial. Lancet.

[39] Khunti K., Wolden M.L., Thorsted B.L., Andersen M., Davies M.J. (2013). Clinical inertia in people with type 2 diabetes: A retrospective cohort study of more than 80,000 people. Diabetes Care.

[40] Cobilinschi C.O., Grădinaru E., Săulescu I., Cârstea N., Caraiola S., Bălănescu A.R., Opriș-Belinski D. (2023). Refractory Takayasu’s Arteritis with Severe Coronary Involvement-Case Report and Literature Review. J. Clin. Med..

[41] Gallwitz B., Dagogo-Jack S., Thieu V., Garcia-Perez L.E., Pavo I., Yu M., Robertson K.E., Zhang N., Giorgino F. (2018). Effect of once-weekly dulaglutide on glycated haemoglobin (HbA1c) and fasting blood glucose in patient subpopulations by gender, duration of diabetes and baseline HbA1c. Diabetes Obes Metab..

[42] Aroda V.R., Bain S.C., Cariou B., Piletič M., Rose L., Axelsen M., Rowe E., DeVries J.H. (2017). Efficacy and safety of once-weekly semaglutide versus once-daily insulin glargine as add-on to metformin (with or without sulfonylureas) in insulin-naive patients with type 2 diabetes (SUSTAIN 4): A randomised, open-label, parallel-group, multicentre, multinational, phase 3a trial. Lancet Diabetes Endocrinol..

[43] Sorli C., Harashima S.I., Tsoukas G.M., Unger J., Karsbøl J.D., Hansen T., Bain S.C. (2017). Efficacy and safety of once-weekly semaglutide monotherapy versus placebo in patients with type 2 diabetes (SUSTAIN 1): A double-blind, randomised, placebo-controlled, parallel-group, multinational, multicentre phase 3a trial. Lancet Diabetes Endocrinol..

[44] Aroda V.R., Ahmann A., Cariou B., Chow F., Davies M.J., Jódar E., Mehta R., Woo V., Lingvay I. (2019). Comparative efficacy, safety, and cardiovascular outcomes with once-weekly subcutaneous semaglutide in the treatment of type 2 diabetes: Insights from the SUSTAIN 1-7 trials. Diabetes Metab..

[45] Jendle J., Grunberger G., Blevins T., Giorgino F., Hietpas R.T., Botros F.T. (2016). Efficacy and safety of dulaglutide in the treatment of type 2 diabetes: A comprehensive review of the dulaglutide clinical data focusing on the AWARD phase 3 clinical trial program. Diabetes Metab. Res. Rev..

[46] Pratley R.E., Aroda V.R., Lingvay I., Lüdemann J., Andreassen C., Navarria A., Viljoen A. (2018). Semaglutide versus dulaglutide in patients with type 2 diabetes (SUSTAIN 7): A randomised, open-label trial. Lancet Diabetes Endocrinol..

[47] Capehorn M.S., Catarig A.-M., Furberg J.K., Janez A., Price H., Tadayon S., Vergès B., Marre M. (2020). Efficacy and safety of once-weekly semaglutide versus exenatide ER in subjects with type 2 diabetes (SUSTAIN 10). Diabetes Metab..

[48] Rodbard H.W., Rosenstock J., Canani L.H., Deerochanawong C., Gumprecht J., Lindberg S.Ø., Lingvay I., Søndergaard A.L., Treppendahl M.B., Montanya E. (2019). Oral semaglutide versus empagliflozin in type 2 diabetes: PIONEER 2 trial. Lancet Diabetes Endocrinol..

[49] Popoviciu M.S., Păduraru L., Yahya G., Metwally K., Cavalu S. (2023). Emerging Role of GLP-1 Agonists in Obesity: A Comprehensive Review of Randomised Controlled Trials. Int. J. Mol. Sci..

[50] OMS (2022). European Regional Obezity Report 2022.

[51] Patti A.M., Giglio R.V., Ciaccio M., Stoian A.P., Salmen T., Bica I.-C., Rangraze I., Tanani M.E., Rizzo M., Rizvi A.A. (2025). New Frontiers in Nutritional and Therapeutic Interventions for Obesity Phenotypes. Medicina.

[52] Elena Mirea L., Cobilinschi C., Grințescu I.M. (2023). Rebranding Nutritional Care for Critically Ill Patients. J. Crit. Care Med. (Univ. De Med. Si Farm. Din Targu-Mures).

[53] Li Y., Gong X., Găman M.A., Hernández-Wolters B., Velu P., Li Y. (2024). The effect of subcutaneous dulaglutide on weight loss in patients with Type 2 diabetes mellitus: Systematic review and meta-analysis of randomized controlled trials. Eur. J. Clin. Investig..

[54] Bade S., Bade S., Sharma G., Bhurtel N., Singh Y., Paudel S., Magar F.P., Chapagain K. (2024). Transformative weight loss with Dulaglutide: A case report of success in a challenging patient profile of an ex-sumo wrestler. Clin. Case Rep..

[55] Kimura T., Kubo M., Takahashi K., Wamata R., Iwamoto Y., Iwamoto H., Katakura Y., Sanada J., Fushimi Y., Shimoda M. (2024). Usefulness of Once-Weekly GLP-1 Receptor Agonist Semaglutide on Glycemic Control in Subjects with Type 2 Diabetes Mellitus: Switching from the Same Class Dulaglutide in a Retrospective Observation Study. J. Diabetes Res..

[56] Reurean-Pintilei D., Potcovaru C.-G., Salmen T., Mititelu-Tartau L., Cinteză D., Lazăr S., Pantea Stoian A., Timar R., Timar B. (2024). Assessment of Cardiovascular Risk Categories and Achievement of Therapeutic Targets in European Patients with Type 2 Diabetes. J. Clin. Med..

[57] Rosenstock J., Klaff L.J., Schwartz S., Northrup J., Holcombe J.H., Wilhelm K., Trautmann M. (2010). Effects of Exenatide and Lifestyle Modification on Body Weight and Glucose Tolerance in Obese Subjects With and Without Pre-Diabetes. Diabetes Care.

[58] Davies M., Pieber T.R., Hartoft-Nielsen M.L., Hansen O.K., Jabbour S., Rosenstock J. (2017). Effect of oral semaglutide compared with placebo and subcutaneous semaglutide on glycemic control in patients with type 2 diabetes: A randomized clinical trial. JAMA.

[59] Husain M., Birkenfeld A.L., Donsmark M., Dungan K., Eliaschewitz F.G., Franco D.R., Jeppesen O.K., Lingvay I., Mosenzon O., Pedersen S.D. (2019). PIONEER 6 Investigators. Oral semaglutide and cardiovascular outcomes in patients with type 2 diabetes. N. Engl. J. Med..

[60] Aroda V.R., Rosenstock J., Terauchi Y., Altuntas Y., Lalic N.M., Morales Villegas E.C., Jeppesen O.K., Christiansen E., Hertz C.L., Haluzík M. (2019). PIONEER 1 Investigators. PIONEER 1: Randomized Clinical Trial of the Efficacy and Safety of Oral Semaglutide Monotherapy in Comparison With Placebo in Patients With Type 2 Diabetes. Diabetes Care.

[61] Mayer C.S., Fontelo P. (2024). Semaglutide use in people with obesity and type 2 diabetes from real-world utilization data: An analysis of the All of US Program. Diabetes Obes. Metab..

[62] Tânţu M.M., Belu E., Man G.M., Manu D., Rogozea L.M., Stocheci C.M., Bălăşoiu A.T., Stănescu C., Domnariu C.D. (2014). Prevalence and histopathological types of skin carcinomas in Arges County, Romania. Rom. J. Morphol. Embryol..

[63] Fadini G.P., Bonora B.M., Ghiani M., Anichini R., Melchionda E., Fattor B., Fazion S., Meregalli G., Giaccari A., Avogaro A. (2024). GLIMPLES study investigators. Oral or injectable semaglutide for the management of type 2 diabetes in routine care: A multicentre observational study comparing matched cohorts. Diabetes Obes. Metab..

[64] Singh A.K., Singh R., Singh A., Misra A. (2024). Efficacy and safety of oral semaglutide in type 2 diabetes: A systematic review of real-world evidence. Diabetes Metab. Syndr..

[65] Sun F., Wu S., Guo S., Yu K., Yang Z., Li L., Zhang Y., Ji L., Zhan S. (2015). Effect of GLP-1 receptor agonists on waist circumference among type 2 diabetes patients: A systematic review and network meta-analysis. Endocrine.

[66] Bohiltea R.E., Zugravu C.A., Nemescu D., Turcan N., Paulet F.P., Gherghiceanu F., Ducu I., Cirstoiu M.M. (2020). Impact of obesity on the prognosis of hypertensive disorders in pregnancy. Exp. Ther. Med..

[67] Vilsbøll T., Christensen M., Junker A.E., Knop F.K., Gluud L.L. (2012). Effects of glucagon-like peptide-1 receptor agonists on weight loss: Systematic review and meta-analyses of randomised controlled trials. BMJ.

[68] Bohiltea R.E., Zugravu C.A., Neacsu A., Navolan D., Berceanu C., Nemescu D., Bodean O., Turcan N., Baros A., Cirstoiu M.M. (2019). The Prevalence of Vitamin D Defficiency and its Obstetrical Effects. Rev. Chim..

[69] Thompson A.M., Trujillo J.M. (2015). Dulaglutide: The newest GLP-1 receptor agonist for the management of type 2 diabetes. Ann. Pharmacother..

[70] Zugravu C., Petra A., Pietroșel V.-A., Mihai B.-M., Mihai D.-A., Bohîlțea R.-E., Tarcea M. (2023). Nutritional Interventions and Lifestyle Changing in Gestational Diabetes Mellitus Prevention: A Narrative Review. Sustainability.

[71] Mauvais-Jarvis F. (2015). Gender differences in glucose homeostasis and diabetes pathophysiology. Diabetes.

[72] Caruso I., Giordano F., Matichecchia I.I., Di Gioia L., Di Molfetta S., Cignarelli A., Sorice G.P., Natalicchio A., Laviola L., Giorgino F. (2025). Effectiveness of GLP-1RA according to different type 2 diabetes phenotypes: A retrospective study. Diabetes Obes. Metab..

[73] Marassi M., Cignarella A., Russo G.T., Nollino L., Strazzabosco M., Marzullo P., Leonetti F., Avogaro A., Consoli A., Fadini G.P. (2025). GLIMPLES study investigators. Sex differences in the weight response to GLP-1RA in people with type 2 diabetes. A long-term longitudinal real-world study. Pharmacol. Res..

